# 5′′-(4-Meth­oxy­benzyl­idene)-1′-(4-meth­oxy­phen­yl)-1′′-methyl-1′,2′,3′,5′,6′,7′,8′,8a′-octa­hydro­dispiro­[acenaphthyl­ene-1,3′-indolizine-2′,3′′-piperidine]-2,4′′(1*H*)-dione

**DOI:** 10.1107/S1600536813001177

**Published:** 2013-01-19

**Authors:** J. Suresh, R. A. Nagalakshmi, S. Sivakumar, R. Ranjith Kumar, P. L. Nilantha Lakshman

**Affiliations:** a Department of Physics, The Madura College, Madurai 625 011, India; b Department of Organic Chemistry, School of Chemistry, Madurai Kamaraj University, Madurai 625 021, India; c Department of Food Science and Technology, University of Ruhuna, Mapalana, Kamburupitiya 81100, Sri Lanka

## Abstract

In the title compound, C_39_H_38_N_2_O_4_, the pyridinone ring adopts a twisted half-chair conformation with the N atom deviating by 0.3304 (1) and with the methyl­ene C atom adjacent to the octa­hydro­indolizine unit deviating by 0.444 (3) Å from the mean plane defined by the other four atoms. In the octa­hydro­indolizine system, the pyrrolidine ring exhibits an envelope conformation, with the fused methyne C atom deviating by 0.6315 (1) Å from the mean plane defined by the other four atoms, and the piperidine ring exhibits a distorted chair conformation, as reflected in the puckering parameters *Q* = 0.568 (4) Å, θ = 1.5 (4) and ϕ = 161 (16)°. In the crystal pairs of weak C—H⋯O inter­actions form centrosymmetric dimers, which are further connected by C—H⋯π inter­actions. The crystal studied was a non-merohedral twin, with a domain ratio of 0.91:0.09.

## Related literature

For general properties of indolizines, see: Weidner *et al.* (1989 ▶); Katritzky *et al.* (1999 ▶); Asano *et al.* (2000 ▶); Gilchrist (2001 ▶); Sarkunam & Nallu (2005 ▶); Tielmann & Hoenke (2006 ▶); Oslund *et al.* (2008 ▶); Vemula *et al.* (2011 ▶); Singh & Mmatli (2011 ▶). For bond lengths and angles in a related structure, see: Suresh *et al.* (2011 ▶). For ring conformation analysis, see: Cremer & Pople (1975 ▶).
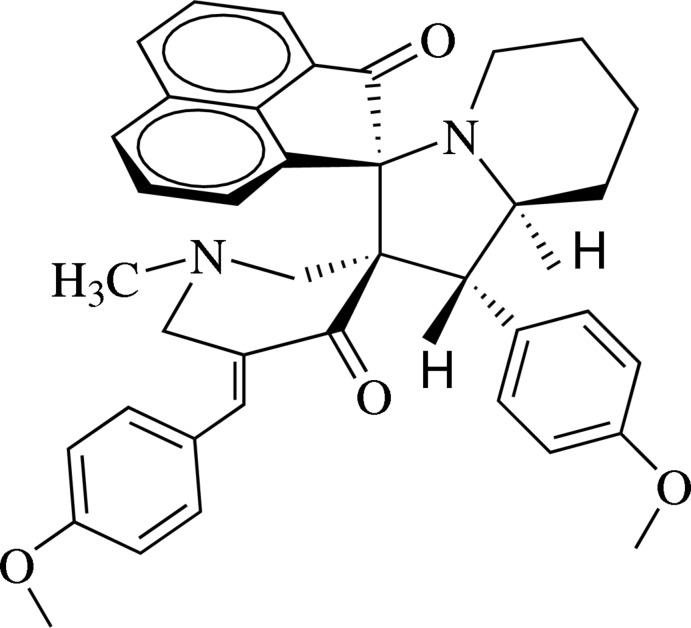


## Experimental

### 

#### Crystal data

C_39_H_38_N_2_O_4_
*M* *_r_* = 598.71
Monoclinic, 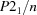

*a* = 8.379 (5) Å
*b* = 16.958 (5) Å
*c* = 22.063 (5) Å
β = 96.605 (5)°
*V* = 3114 (2) Å^3^
*Z* = 4
Mo *K*α radiation
μ = 0.08 mm^−1^
*T* = 293 K
0.21 × 0.19 × 0.18 mm

#### Data collection

Bruker Kappa APEXII diffractometer
Absorption correction: multi-scan (*SADABS*; Sheldrick, 1996 ▶) *T*_min_ = 0.967, *T*_max_ = 0.974
5537 measured reflections
5537 independent reflections
3900 reflections with *I* > 2σ(*I*)

#### Refinement

*R*[*F* ^2^ > 2σ(*F* ^2^)] = 0.066
*wR*(*F* ^2^) = 0.183
*S* = 1.05
5537 reflections
407 parameters
H-atom parameters constrained
Δρ_max_ = 0.44 e Å^−3^
Δρ_min_ = −0.23 e Å^−3^

### 

Data collection: *APEX2* (Bruker, 2004 ▶); cell refinement: *SAINT* (Bruker, 2004 ▶); data reduction: *SAINT*; program(s) used to solve structure: *SHELXS97* (Sheldrick, 2008 ▶); program(s) used to refine structure: *SHELXL97* (Sheldrick, 2008 ▶); molecular graphics: *PLATON* (Spek, 2009 ▶); software used to prepare material for publication: *SHELXL97*.

## Supplementary Material

Crystal structure: contains datablock(s) global, I. DOI: 10.1107/S1600536813001177/bh2471sup1.cif

Structure factors: contains datablock(s) I. DOI: 10.1107/S1600536813001177/bh2471Isup2.hkl

Additional supplementary materials:  crystallographic information; 3D view; checkCIF report

## Figures and Tables

**Table 1:** Hydrogen-bond geometry (Å, °) *Cg*1 is the centroid of the benzene ring (C52–C57) in the phenyl­methyl­idene group.

*D*—H⋯*A*	*D*—H	H⋯*A*	*D*⋯*A*	*D*—H⋯*A*
C77—H77*A*⋯O2^i^	0.96	2.54	3.418 (5)	152
C58—H58*C*⋯*Cg*1^ii^	0.96	2.93	3.822 (5)	156

Symmetry codes: (i) 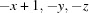; (ii) 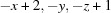.

**Fig. 1.**
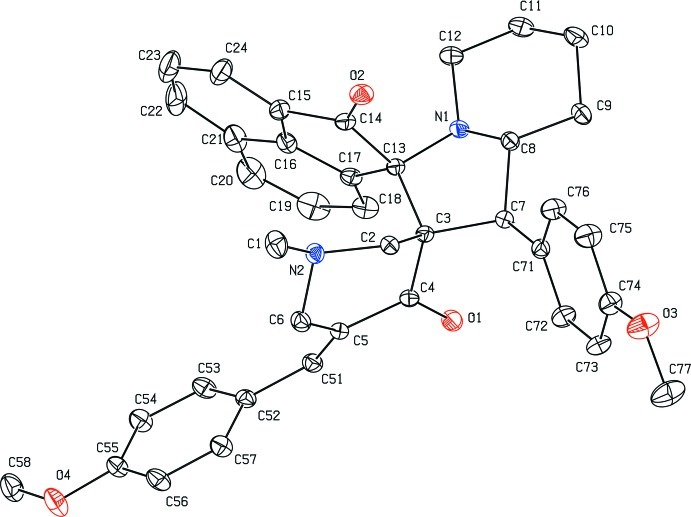
The molecular structure of the title compound, showing 50% probability displacement ellipsoids.

**Fig. 2.**
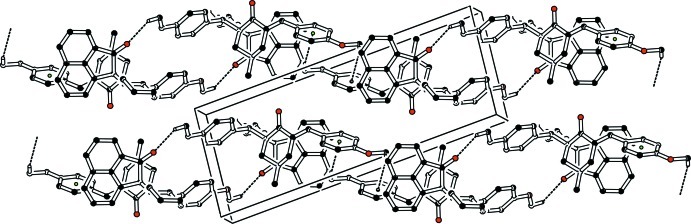
Partial packing diagram showing C—H···O and C—H···π interactions.

## supplementary crystallographic information

### Comment

Indolizines are used as dyes (Weidner *et al.*, 1989), pharmaceuticals (Singh & Mmatli, 2011), and spectroscopic sensitizers (Katritzky *et al.*, 1999; Sarkunam & Nallu, 2005; Gilchrist, 2001; Vemula *et al.*, 2011). Indolizines, both synthetic and natural, have also been ascribed with a number of useful biological activities (Asano *et al.*, 2000; Tielmann & Hoenke, 2006; Oslund *et al.*, 2008) such as antibacterial, antiviral, CNS depressants, anti-HIV, anti-cancer, and have been used for treating cardiovascular ailments. In view of its medicinal importance we report the crystal structure of the title compound.

In the title compound (Fig. 1), the central pyridinone ring with the methyl substituent in an equatorial position, adopts twisted half chair conformation with atoms N2 and C2 deviating by 0.3304 (1) and -0.444 (3) Å respectively, from the mean plane defined by other atoms C3/C4/C5/C6. The O1 atom is deviating by -0.4117 (1) Å from the above mean plane. The sum of bond angles around N2, 332 (9)°, indicates a pyramidal geometry. In the fused system, the pyrrolidine ring adopts the twisted envelope conformation with C8 atom at the flap deviating by -0.6315 (1) Å from the mean plane defined by other atoms C7/C3/C13/N1, and this orientation may be due to the inter-molecular C7— H7A···O2 interaction. In the fused system, the piperidine ring adopts a slightly distorted chair conformation as evident from the puckering parameters *Q* = 0.568 (4) Å, θ = 1.5 (4)° and φ = 161 (16)° (Cremer & Pople, 1975). The twist of the 4-methoxy benzene ring (C52 to C57) with respect to the spiro junction is denoted by the torsion angle C5—C51—C52—C57 = -46.4 (5)°. The dihedral angles between the mean plane of the pyridinone ring, defined by atoms C3/C4/C5/C6 and 4-methoxy benzene rings are 85.58 (1) and 61.91 (1)°. The carbonyl bond lengths, C4═O1 and C14═02 [1.217 (4) Å for both], are somewhat long, due to C—H···O contacts. Although atoms C8, C12, C13 attached to the atom N1, are all *sp*^3^ hybridized, their different environments cause differences in bond lengths [N1—C12: 1.466 (4) Å, N1—C8: 1.453 (4) Å, and N1—C13: 1.466 (4) Å] and in the bond angles [C12—N1—C13: 116.9 (3)°, C13—N1—C8: 107.7 (2)° and C12—N1—C8: 114.3 (3)°]. The methoxy groups substituted at the phenyl rings are nearly coplanar, as it can be seen from the torsion angles C77—O3—C74—C73 = 2.5 (6)° and C58—O4—C55—C54 = -3.9 (6)°. The C—C bond lengths and C—C—C angles in the acenaphthylene group compare with those of related structures (Suresh *et al.*, 2011).

The structure features weak intra-molecular interactions and inter-molecular interactions. A weak inter-molecular interaction, viz C77—H77A···O2, generates a ring motif *R*^2^_2_(25), forming centrosymmetric dimers, and these dimers are interconnected by C—H···π interactions (Fig. 2), involving the benzene ring C52···C57.

### Experimental

A mixture of 1-methyl-3,5-bis[(*E*)-4-methoxyphenyl-methylidene]tetrahydro-4(1*H*)-pyridinone (1 mmol), acenaphthenequinone (1 mmol) and piperidine-2-carboxylic acid (1 mmol) was dissolved in isopropyl alcohol (15 ml) and refluxed for 60 min. After completion of the reaction (TLC), the mixture was poured into water (50 ml), the precipitated solid was filtered and washed with water (100 ml) to obtain pure yellow solid. Melting point: 520 K, yield: 94%.

### Refinement

H atoms were placed at calculated positions and allowed to ride on their carrier atoms with C—H = 0.93–0.98 Å; *U*_iso_ = 1.2*U*_eq_(C) for CH_2_ and CH groups, and *U*_iso_ = 1.5*U*_eq_(C) for CH_3_ groups. The investigated crystal was a non-merohedral twin, with a twin law [-1 0 0, 0 -1 0, 0.606 0 1] and with a ratio of twin components of 0.91:09, corresponding to a 2-fold rotation about (0 0 1), as determined with the TwinRotMat option of *PLATON* (Spek, 2009). The final refinement was carried out against a detwinned set. Three strongly deviated reflections were omitted in the final refinement.

### Crystal data

**Table:**

C_39_H_38_N_2_O_4_	*F*(000) = 1272
*M**_r_* = 598.71	*D*_x_ = 1.277 Mg m^−^^3^
Monoclinic, *P*2_1_/*n*	Melting point: 520 K
Hall symbol: -P 2yn	Mo *K*α radiation, λ = 0.71073 Å
*a* = 8.379 (5) Å	Cell parameters from 2000 reflections
*b* = 16.958 (5) Å	θ = 2–31°
*c* = 22.063 (5) Å	µ = 0.08 mm^−^^1^
β = 96.605 (5)°	*T* = 293 K
*V* = 3114 (2) Å^3^	Block, yellow
*Z* = 4	0.21 × 0.19 × 0.18 mm

### Data collection

**Table:**

Bruker Kappa APEXII diffractometer	5537 independent reflections
Radiation source: fine-focus sealed tube	3900 reflections with *I* > 2σ(*I*)
Graphite monochromator	*R*_int_ = 0.000
Detector resolution: 0 pixels mm^-1^	θ_max_ = 25.2°, θ_min_ = 1.5°
ω and φ scans	*h* = −9→9
Absorption correction: multi-scan (*SADABS*; Sheldrick, 1996)	*k* = −20→20
*T*_min_ = 0.967, *T*_max_ = 0.974	*l* = −11→26
5537 measured reflections	

### Refinement

**Table:**

Refinement on *F*^2^	Primary atom site location: structure-invariant direct methods
Least-squares matrix: full	Secondary atom site location: difference Fourier map
*R*[*F*^2^ > 2σ(*F*^2^)] = 0.066	Hydrogen site location: inferred from neighbouring sites
*wR*(*F*^2^) = 0.183	H-atom parameters constrained
*S* = 1.05	*w* = 1/[σ^2^(*F*_o_^2^) + (0.0636*P*)^2^ + 4.5987*P*] where *P* = (*F*_o_^2^ + 2*F*_c_^2^)/3
5537 reflections	(Δ/σ)_max_ < 0.001
407 parameters	Δρ_max_ = 0.44 e Å^−^^3^
0 restraints	Δρ_min_ = −0.23 e Å^−^^3^
0 constraints	

### Fractional atomic coordinates and isotropic or equivalent isotropic displacement parameters (Å^2^)

**Table:**

	*x*	*y*	*z*	*U*_iso_*/*U*_eq_	
C1	0.8861 (4)	0.0366 (3)	0.20452 (18)	0.0536 (10)	
H1A	0.9729	0.0311	0.2366	0.080*	
H1B	0.9130	0.0762	0.1763	0.080*	
H1C	0.8681	−0.0128	0.1836	0.080*	
C2	0.6039 (4)	0.06949 (18)	0.18388 (14)	0.0311 (7)	
H2A	0.5635	0.0181	0.1702	0.037*	
H2B	0.6376	0.0972	0.1490	0.037*	
C3	0.4716 (4)	0.11596 (16)	0.20994 (13)	0.0278 (7)	
C4	0.4221 (4)	0.06915 (17)	0.26417 (14)	0.0311 (7)	
C5	0.5514 (4)	0.02671 (17)	0.30318 (14)	0.0306 (7)	
C6	0.7001 (4)	0.00261 (19)	0.27528 (15)	0.0376 (8)	
H6A	0.7894	−0.0024	0.3072	0.045*	
H6B	0.6825	−0.0484	0.2558	0.045*	
C7	0.3264 (4)	0.13394 (17)	0.16133 (14)	0.0298 (7)	
H7	0.2299	0.1319	0.1825	0.036*	
C8	0.3503 (4)	0.21966 (17)	0.14482 (14)	0.0317 (7)	
H8	0.4356	0.2236	0.1181	0.038*	
C9	0.2017 (4)	0.26274 (19)	0.11600 (17)	0.0426 (9)	
H9A	0.1139	0.2546	0.1404	0.051*	
H9B	0.1697	0.2419	0.0755	0.051*	
C10	0.2372 (5)	0.3506 (2)	0.11197 (19)	0.0539 (10)	
H10A	0.1402	0.3784	0.0960	0.065*	
H10B	0.3174	0.3591	0.0843	0.065*	
C11	0.2983 (5)	0.3829 (2)	0.17483 (19)	0.0546 (10)	
H11A	0.3261	0.4381	0.1713	0.066*	
H11B	0.2139	0.3791	0.2013	0.066*	
C12	0.4441 (5)	0.33755 (18)	0.20268 (18)	0.0458 (9)	
H12A	0.5328	0.3459	0.1788	0.055*	
H12B	0.4762	0.3563	0.2438	0.055*	
C13	0.5304 (4)	0.20110 (17)	0.23384 (14)	0.0281 (7)	
C14	0.7028 (4)	0.22267 (18)	0.21664 (16)	0.0353 (8)	
C15	0.8039 (4)	0.24767 (19)	0.27284 (16)	0.0398 (8)	
C16	0.7076 (4)	0.24392 (19)	0.32061 (15)	0.0383 (8)	
C17	0.5508 (4)	0.21718 (17)	0.30216 (14)	0.0344 (8)	
C18	0.4439 (5)	0.2155 (2)	0.34435 (16)	0.0465 (9)	
H18	0.3378	0.2005	0.3333	0.056*	
C19	0.4977 (7)	0.2370 (3)	0.40524 (19)	0.0670 (13)	
H19	0.4263	0.2338	0.4345	0.080*	
C20	0.6504 (7)	0.2624 (3)	0.42254 (19)	0.0709 (14)	
H20	0.6805	0.2760	0.4631	0.085*	
C21	0.7631 (5)	0.2684 (2)	0.38039 (18)	0.0554 (11)	
C22	0.9208 (6)	0.2975 (3)	0.3887 (2)	0.0740 (15)	
H22	0.9631	0.3149	0.4272	0.089*	
C23	1.0143 (6)	0.3010 (3)	0.3419 (3)	0.0756 (15)	
H23	1.1184	0.3206	0.3494	0.091*	
C24	0.9574 (5)	0.2758 (2)	0.2829 (2)	0.0583 (11)	
H24	1.0222	0.2782	0.2514	0.070*	
C51	0.5327 (4)	0.01730 (18)	0.36210 (15)	0.0362 (8)	
H51	0.4389	0.0382	0.3743	0.043*	
C52	0.6398 (4)	−0.02137 (18)	0.40965 (14)	0.0341 (7)	
C53	0.6721 (4)	0.0136 (2)	0.46697 (15)	0.0427 (9)	
H53	0.6244	0.0617	0.4741	0.051*	
C54	0.7726 (4)	−0.0208 (2)	0.51350 (16)	0.0456 (9)	
H54	0.7935	0.0045	0.5510	0.055*	
C55	0.8418 (4)	−0.0929 (2)	0.50414 (15)	0.0399 (8)	
C56	0.8081 (5)	−0.1299 (2)	0.44809 (16)	0.0469 (9)	
H56	0.8526	−0.1791	0.4418	0.056*	
C57	0.7096 (4)	−0.0948 (2)	0.40169 (15)	0.0409 (8)	
H57	0.6892	−0.1204	0.3643	0.049*	
C58	0.9814 (5)	−0.0940 (3)	0.60414 (18)	0.0623 (11)	
H58A	1.0508	−0.1275	0.6305	0.093*	
H58B	0.8855	−0.0833	0.6226	0.093*	
H58C	1.0357	−0.0454	0.5977	0.093*	
C71	0.2988 (4)	0.07706 (18)	0.10796 (14)	0.0333 (7)	
C72	0.2147 (4)	0.0085 (2)	0.11315 (17)	0.0442 (9)	
H72	0.1765	−0.0032	0.1501	0.053*	
C73	0.1844 (4)	−0.0451 (2)	0.06395 (17)	0.0473 (9)	
H73	0.1277	−0.0915	0.0684	0.057*	
C74	0.2402 (5)	−0.0272 (2)	0.00964 (16)	0.0458 (9)	
C75	0.3242 (5)	0.0405 (2)	0.00330 (17)	0.0547 (10)	
H75	0.3615	0.0522	−0.0338	0.066*	
C76	0.3539 (5)	0.0915 (2)	0.05173 (15)	0.0474 (9)	
H76	0.4125	0.1372	0.0468	0.057*	
C77	0.1333 (6)	−0.1444 (3)	−0.0375 (2)	0.0757 (14)	
H77A	0.1286	−0.1720	−0.0757	0.114*	
H77B	0.0264	−0.1320	−0.0290	0.114*	
H77C	0.1849	−0.1770	−0.0055	0.114*	
N1	0.4057 (3)	0.25330 (14)	0.20408 (12)	0.0333 (6)	
N2	0.7402 (3)	0.06004 (16)	0.23053 (12)	0.0338 (6)	
O1	0.2835 (3)	0.06822 (14)	0.27573 (11)	0.0433 (6)	
O2	0.7389 (3)	0.22681 (14)	0.16484 (11)	0.0443 (6)	
O3	0.2198 (4)	−0.07557 (17)	−0.04099 (12)	0.0668 (8)	
O4	0.9405 (4)	−0.13225 (16)	0.54736 (11)	0.0606 (8)	

### Atomic displacement parameters (Å^2^)

**Table:**

	*U*^11^	*U*^22^	*U*^33^	*U*^12^	*U*^13^	*U*^23^
C1	0.037 (2)	0.073 (3)	0.052 (2)	0.0131 (19)	0.0120 (18)	0.008 (2)
C2	0.0320 (17)	0.0306 (16)	0.0306 (17)	0.0024 (13)	0.0036 (14)	0.0017 (13)
C3	0.0288 (16)	0.0232 (15)	0.0315 (17)	−0.0002 (12)	0.0031 (13)	0.0003 (12)
C4	0.0341 (19)	0.0236 (15)	0.0353 (18)	−0.0002 (13)	0.0026 (14)	−0.0006 (13)
C5	0.0313 (17)	0.0267 (16)	0.0333 (18)	−0.0016 (13)	0.0023 (14)	0.0048 (13)
C6	0.0359 (18)	0.0354 (18)	0.0414 (19)	0.0065 (14)	0.0033 (15)	0.0023 (15)
C7	0.0302 (17)	0.0280 (16)	0.0309 (17)	−0.0004 (13)	0.0023 (13)	0.0023 (13)
C8	0.0337 (17)	0.0292 (16)	0.0315 (17)	0.0021 (13)	0.0006 (14)	0.0010 (13)
C9	0.044 (2)	0.0373 (19)	0.045 (2)	0.0072 (15)	−0.0030 (17)	0.0064 (16)
C10	0.055 (2)	0.037 (2)	0.069 (3)	0.0105 (17)	0.004 (2)	0.0163 (19)
C11	0.062 (3)	0.0266 (18)	0.074 (3)	0.0054 (17)	0.001 (2)	0.0048 (18)
C12	0.054 (2)	0.0239 (17)	0.058 (2)	0.0002 (15)	0.0003 (18)	−0.0017 (16)
C13	0.0309 (17)	0.0245 (15)	0.0288 (16)	−0.0019 (12)	0.0037 (13)	0.0003 (12)
C14	0.0384 (19)	0.0261 (16)	0.041 (2)	−0.0029 (13)	0.0042 (16)	0.0021 (14)
C15	0.039 (2)	0.0330 (17)	0.045 (2)	−0.0019 (15)	−0.0007 (16)	−0.0029 (15)
C16	0.050 (2)	0.0287 (17)	0.0345 (19)	0.0069 (15)	−0.0043 (16)	−0.0051 (14)
C17	0.045 (2)	0.0243 (15)	0.0340 (18)	0.0022 (14)	0.0065 (15)	−0.0016 (13)
C18	0.058 (2)	0.040 (2)	0.044 (2)	0.0043 (17)	0.0172 (18)	−0.0053 (16)
C19	0.103 (4)	0.060 (3)	0.042 (2)	0.009 (3)	0.027 (3)	−0.012 (2)
C20	0.105 (4)	0.069 (3)	0.036 (2)	0.015 (3)	−0.003 (3)	−0.021 (2)
C21	0.068 (3)	0.047 (2)	0.047 (2)	0.014 (2)	−0.010 (2)	−0.0151 (18)
C22	0.071 (3)	0.069 (3)	0.072 (3)	0.012 (2)	−0.033 (3)	−0.035 (3)
C23	0.048 (3)	0.066 (3)	0.106 (4)	−0.001 (2)	−0.022 (3)	−0.029 (3)
C24	0.041 (2)	0.056 (2)	0.075 (3)	−0.0083 (18)	−0.004 (2)	−0.012 (2)
C51	0.0338 (18)	0.0321 (17)	0.043 (2)	0.0026 (14)	0.0065 (15)	0.0054 (15)
C52	0.0367 (18)	0.0343 (17)	0.0323 (18)	−0.0008 (14)	0.0078 (14)	0.0062 (14)
C53	0.056 (2)	0.0338 (18)	0.039 (2)	0.0095 (16)	0.0091 (17)	0.0011 (15)
C54	0.059 (2)	0.041 (2)	0.036 (2)	0.0049 (17)	0.0020 (17)	−0.0025 (16)
C55	0.047 (2)	0.0382 (18)	0.0340 (19)	0.0051 (16)	0.0029 (16)	0.0044 (15)
C56	0.063 (2)	0.0333 (18)	0.044 (2)	0.0143 (17)	0.0041 (18)	0.0013 (16)
C57	0.052 (2)	0.0372 (18)	0.0331 (18)	0.0032 (16)	0.0031 (16)	−0.0021 (15)
C58	0.072 (3)	0.064 (3)	0.047 (2)	0.010 (2)	−0.012 (2)	0.002 (2)
C71	0.0312 (17)	0.0299 (17)	0.0378 (19)	0.0024 (13)	−0.0006 (14)	−0.0020 (14)
C72	0.047 (2)	0.0404 (19)	0.047 (2)	−0.0053 (16)	0.0103 (17)	−0.0066 (16)
C73	0.051 (2)	0.0358 (19)	0.056 (2)	−0.0126 (16)	0.0071 (19)	−0.0051 (17)
C74	0.050 (2)	0.049 (2)	0.038 (2)	−0.0063 (17)	0.0061 (17)	−0.0034 (17)
C75	0.074 (3)	0.055 (2)	0.036 (2)	−0.012 (2)	0.010 (2)	−0.0026 (17)
C76	0.063 (2)	0.044 (2)	0.035 (2)	−0.0118 (18)	0.0069 (18)	−0.0020 (16)
C77	0.105 (4)	0.063 (3)	0.062 (3)	−0.034 (3)	0.018 (3)	−0.029 (2)
N1	0.0384 (15)	0.0228 (13)	0.0376 (15)	0.0010 (11)	−0.0005 (12)	−0.0008 (11)
N2	0.0286 (14)	0.0388 (15)	0.0342 (15)	0.0017 (11)	0.0047 (12)	0.0030 (12)
O1	0.0317 (14)	0.0499 (15)	0.0494 (15)	0.0019 (11)	0.0092 (11)	0.0153 (11)
O2	0.0479 (15)	0.0467 (14)	0.0401 (15)	−0.0058 (11)	0.0128 (12)	0.0073 (11)
O3	0.092 (2)	0.0649 (19)	0.0465 (16)	−0.0261 (16)	0.0200 (15)	−0.0175 (14)
O4	0.079 (2)	0.0550 (16)	0.0431 (15)	0.0232 (14)	−0.0124 (14)	−0.0004 (13)

### Geometric parameters (Å, º)

**Table:**

C1—N2	1.464 (4)	C17—C18	1.365 (5)
C1—H1A	0.9600	C18—C19	1.415 (6)
C1—H1B	0.9600	C18—H18	0.9300
C1—H1C	0.9600	C19—C20	1.362 (7)
C2—N2	1.456 (4)	C19—H19	0.9300
C2—C3	1.526 (4)	C20—C21	1.403 (7)
C2—H2A	0.9700	C20—H20	0.9300
C2—H2B	0.9700	C21—C22	1.403 (7)
C3—C4	1.533 (4)	C22—C23	1.367 (7)
C3—C7	1.557 (4)	C22—H22	0.9300
C3—C13	1.595 (4)	C23—C24	1.401 (6)
C4—O1	1.217 (4)	C23—H23	0.9300
C4—C5	1.489 (4)	C24—H24	0.9300
C5—C51	1.337 (4)	C51—C52	1.456 (4)
C5—C6	1.509 (5)	C51—H51	0.9300
C6—N2	1.453 (4)	C52—C53	1.395 (5)
C6—H6A	0.9700	C52—C57	1.396 (5)
C6—H6B	0.9700	C53—C54	1.380 (5)
C7—C8	1.517 (4)	C53—H53	0.9300
C7—C71	1.519 (4)	C54—C55	1.379 (5)
C7—H7	0.9800	C54—H54	0.9300
C8—N1	1.453 (4)	C55—O4	1.363 (4)
C8—C9	1.518 (4)	C55—C56	1.386 (5)
C8—H8	0.9800	C56—C57	1.375 (5)
C9—C10	1.524 (5)	C56—H56	0.9300
C9—H9A	0.9700	C57—H57	0.9300
C9—H9B	0.9700	C58—O4	1.417 (4)
C10—C11	1.524 (5)	C58—H58A	0.9600
C10—H10A	0.9700	C58—H58B	0.9600
C10—H10B	0.9700	C58—H58C	0.9600
C11—C12	1.512 (5)	C71—C72	1.372 (5)
C11—H11A	0.9700	C71—C76	1.394 (5)
C11—H11B	0.9700	C72—C73	1.415 (5)
C12—N1	1.466 (4)	C72—H72	0.9300
C12—H12A	0.9700	C73—C74	1.370 (5)
C12—H12B	0.9700	C73—H73	0.9300
C13—N1	1.466 (4)	C74—C75	1.362 (5)
C13—C17	1.522 (4)	C74—O3	1.381 (4)
C13—C14	1.579 (5)	C75—C76	1.375 (5)
C14—O2	1.217 (4)	C75—H75	0.9300
C14—C15	1.481 (5)	C76—H76	0.9300
C15—C24	1.366 (5)	C77—O3	1.381 (5)
C15—C16	1.400 (5)	C77—H77A	0.9600
C16—C17	1.405 (5)	C77—H77B	0.9600
C16—C21	1.410 (5)	C77—H77C	0.9600
			
N2—C1—H1A	109.5	C18—C17—C13	131.6 (3)
N2—C1—H1B	109.5	C16—C17—C13	109.9 (3)
H1A—C1—H1B	109.5	C17—C18—C19	118.7 (4)
N2—C1—H1C	109.5	C17—C18—H18	120.7
H1A—C1—H1C	109.5	C19—C18—H18	120.7
H1B—C1—H1C	109.5	C20—C19—C18	122.3 (4)
N2—C2—C3	109.6 (2)	C20—C19—H19	118.9
N2—C2—H2A	109.8	C18—C19—H19	118.9
C3—C2—H2A	109.8	C19—C20—C21	121.3 (4)
N2—C2—H2B	109.8	C19—C20—H20	119.4
C3—C2—H2B	109.8	C21—C20—H20	119.4
H2A—C2—H2B	108.2	C22—C21—C20	129.4 (4)
C2—C3—C4	107.5 (2)	C22—C21—C16	115.4 (4)
C2—C3—C7	112.7 (2)	C20—C21—C16	115.1 (4)
C4—C3—C7	112.3 (2)	C23—C22—C21	122.0 (4)
C2—C3—C13	112.5 (2)	C23—C22—H22	119.0
C4—C3—C13	108.2 (2)	C21—C22—H22	119.0
C7—C3—C13	103.7 (2)	C22—C23—C24	121.7 (4)
O1—C4—C5	121.4 (3)	C22—C23—H23	119.2
O1—C4—C3	121.4 (3)	C24—C23—H23	119.2
C5—C4—C3	117.2 (3)	C15—C24—C23	118.1 (4)
C51—C5—C4	117.4 (3)	C15—C24—H24	121.0
C51—C5—C6	124.0 (3)	C23—C24—H24	121.0
C4—C5—C6	118.4 (3)	C5—C51—C52	128.6 (3)
N2—C6—C5	111.2 (3)	C5—C51—H51	115.7
N2—C6—H6A	109.4	C52—C51—H51	115.7
C5—C6—H6A	109.4	C53—C52—C57	116.9 (3)
N2—C6—H6B	109.4	C53—C52—C51	120.1 (3)
C5—C6—H6B	109.4	C57—C52—C51	123.0 (3)
H6A—C6—H6B	108.0	C54—C53—C52	122.2 (3)
C8—C7—C71	115.8 (3)	C54—C53—H53	118.9
C8—C7—C3	103.8 (2)	C52—C53—H53	118.9
C71—C7—C3	116.5 (2)	C55—C54—C53	119.6 (3)
C8—C7—H7	106.7	C55—C54—H54	120.2
C71—C7—H7	106.7	C53—C54—H54	120.2
C3—C7—H7	106.7	O4—C55—C54	124.1 (3)
N1—C8—C7	101.4 (2)	O4—C55—C56	116.6 (3)
N1—C8—C9	110.5 (3)	C54—C55—C56	119.3 (3)
C7—C8—C9	115.9 (3)	C57—C56—C55	120.7 (3)
N1—C8—H8	109.6	C57—C56—H56	119.6
C7—C8—H8	109.6	C55—C56—H56	119.6
C9—C8—H8	109.6	C56—C57—C52	121.2 (3)
C8—C9—C10	109.8 (3)	C56—C57—H57	119.4
C8—C9—H9A	109.7	C52—C57—H57	119.4
C10—C9—H9A	109.7	O4—C58—H58A	109.5
C8—C9—H9B	109.7	O4—C58—H58B	109.5
C10—C9—H9B	109.7	H58A—C58—H58B	109.5
H9A—C9—H9B	108.2	O4—C58—H58C	109.5
C9—C10—C11	110.2 (3)	H58A—C58—H58C	109.5
C9—C10—H10A	109.6	H58B—C58—H58C	109.5
C11—C10—H10A	109.6	C72—C71—C76	116.7 (3)
C9—C10—H10B	109.6	C72—C71—C7	120.5 (3)
C11—C10—H10B	109.6	C76—C71—C7	122.8 (3)
H10A—C10—H10B	108.1	C71—C72—C73	121.9 (3)
C12—C11—C10	111.0 (3)	C71—C72—H72	119.0
C12—C11—H11A	109.4	C73—C72—H72	119.0
C10—C11—H11A	109.4	C74—C73—C72	118.6 (3)
C12—C11—H11B	109.4	C74—C73—H73	120.7
C10—C11—H11B	109.4	C72—C73—H73	120.7
H11A—C11—H11B	108.0	C75—C74—C73	120.6 (3)
N1—C12—C11	109.6 (3)	C75—C74—O3	115.8 (3)
N1—C12—H12A	109.8	C73—C74—O3	123.6 (3)
C11—C12—H12A	109.8	C74—C75—C76	120.0 (4)
N1—C12—H12B	109.8	C74—C75—H75	120.0
C11—C12—H12B	109.8	C76—C75—H75	120.0
H12A—C12—H12B	108.2	C75—C76—C71	122.2 (3)
N1—C13—C17	109.2 (2)	C75—C76—H76	118.9
N1—C13—C14	112.2 (2)	C71—C76—H76	118.9
C17—C13—C14	101.2 (2)	O3—C77—H77A	109.5
N1—C13—C3	102.8 (2)	O3—C77—H77B	109.5
C17—C13—C3	119.0 (2)	H77A—C77—H77B	109.5
C14—C13—C3	112.7 (2)	O3—C77—H77C	109.5
O2—C14—C15	126.1 (3)	H77A—C77—H77C	109.5
O2—C14—C13	124.9 (3)	H77B—C77—H77C	109.5
C15—C14—C13	108.4 (3)	C8—N1—C13	107.7 (2)
C24—C15—C16	120.5 (3)	C8—N1—C12	114.3 (3)
C24—C15—C14	132.3 (4)	C13—N1—C12	116.9 (3)
C16—C15—C14	107.1 (3)	C6—N2—C2	109.3 (2)
C15—C16—C17	113.4 (3)	C6—N2—C1	110.7 (3)
C15—C16—C21	122.3 (3)	C2—N2—C1	112.0 (3)
C17—C16—C21	124.2 (3)	C77—O3—C74	118.3 (3)
C18—C17—C16	118.3 (3)	C55—O4—C58	117.4 (3)
			
N2—C2—C3—C4	−60.0 (3)	C13—C17—C18—C19	−177.2 (3)
N2—C2—C3—C7	175.8 (2)	C17—C18—C19—C20	2.8 (6)
N2—C2—C3—C13	58.9 (3)	C18—C19—C20—C21	−0.1 (7)
C2—C3—C4—O1	−145.1 (3)	C19—C20—C21—C22	175.7 (4)
C7—C3—C4—O1	−20.6 (4)	C19—C20—C21—C16	−2.1 (6)
C13—C3—C4—O1	93.2 (3)	C15—C16—C21—C22	0.6 (5)
C2—C3—C4—C5	36.6 (3)	C17—C16—C21—C22	−176.4 (3)
C7—C3—C4—C5	161.1 (3)	C15—C16—C21—C20	178.6 (3)
C13—C3—C4—C5	−85.1 (3)	C17—C16—C21—C20	1.7 (5)
O1—C4—C5—C51	−28.4 (4)	C20—C21—C22—C23	−178.3 (5)
C3—C4—C5—C51	149.9 (3)	C16—C21—C22—C23	−0.6 (6)
O1—C4—C5—C6	156.2 (3)	C21—C22—C23—C24	0.1 (7)
C3—C4—C5—C6	−25.5 (4)	C16—C15—C24—C23	−0.3 (6)
C51—C5—C6—N2	−141.0 (3)	C14—C15—C24—C23	174.5 (4)
C4—C5—C6—N2	34.1 (4)	C22—C23—C24—C15	0.3 (7)
C2—C3—C7—C8	−102.3 (3)	C4—C5—C51—C52	−179.9 (3)
C4—C3—C7—C8	136.2 (3)	C6—C5—C51—C52	−4.8 (5)
C13—C3—C7—C8	19.6 (3)	C5—C51—C52—C53	136.0 (4)
C2—C3—C7—C71	26.3 (4)	C5—C51—C52—C57	−46.4 (5)
C4—C3—C7—C71	−95.3 (3)	C57—C52—C53—C54	2.1 (5)
C13—C3—C7—C71	148.2 (3)	C51—C52—C53—C54	179.9 (3)
C71—C7—C8—N1	−168.6 (3)	C52—C53—C54—C55	−1.3 (6)
C3—C7—C8—N1	−39.6 (3)	C53—C54—C55—O4	−179.0 (3)
C71—C7—C8—C9	71.8 (4)	C53—C54—C55—C56	−0.4 (6)
C3—C7—C8—C9	−159.2 (3)	O4—C55—C56—C57	180.0 (3)
N1—C8—C9—C10	55.9 (4)	C54—C55—C56—C57	1.3 (6)
C7—C8—C9—C10	170.5 (3)	C55—C56—C57—C52	−0.5 (6)
C8—C9—C10—C11	−55.6 (4)	C53—C52—C57—C56	−1.2 (5)
C9—C10—C11—C12	56.0 (4)	C51—C52—C57—C56	−178.9 (3)
C10—C11—C12—N1	−55.1 (4)	C8—C7—C71—C72	−153.6 (3)
C2—C3—C13—N1	129.2 (3)	C3—C7—C71—C72	83.9 (4)
C4—C3—C13—N1	−112.2 (3)	C8—C7—C71—C76	25.4 (4)
C7—C3—C13—N1	7.2 (3)	C3—C7—C71—C76	−97.1 (4)
C2—C3—C13—C17	−110.0 (3)	C76—C71—C72—C73	−0.2 (5)
C4—C3—C13—C17	8.5 (4)	C7—C71—C72—C73	178.8 (3)
C7—C3—C13—C17	127.9 (3)	C71—C72—C73—C74	−0.4 (6)
C2—C3—C13—C14	8.2 (3)	C72—C73—C74—C75	0.5 (6)
C4—C3—C13—C14	126.8 (3)	C72—C73—C74—O3	178.9 (4)
C7—C3—C13—C14	−113.8 (3)	C73—C74—C75—C76	0.1 (6)
N1—C13—C14—O2	−55.5 (4)	O3—C74—C75—C76	−178.5 (4)
C17—C13—C14—O2	−171.8 (3)	C74—C75—C76—C71	−0.8 (6)
C3—C13—C14—O2	60.0 (4)	C72—C71—C76—C75	0.8 (5)
N1—C13—C14—C15	116.5 (3)	C7—C71—C76—C75	−178.2 (3)
C17—C13—C14—C15	0.3 (3)	C7—C8—N1—C13	46.8 (3)
C3—C13—C14—C15	−128.0 (3)	C9—C8—N1—C13	170.2 (3)
O2—C14—C15—C24	−4.2 (6)	C7—C8—N1—C12	178.5 (3)
C13—C14—C15—C24	−176.1 (4)	C9—C8—N1—C12	−58.1 (4)
O2—C14—C15—C16	171.1 (3)	C17—C13—N1—C8	−160.8 (2)
C13—C14—C15—C16	−0.8 (3)	C14—C13—N1—C8	87.8 (3)
C24—C15—C16—C17	177.1 (3)	C3—C13—N1—C8	−33.5 (3)
C14—C15—C16—C17	1.1 (4)	C17—C13—N1—C12	69.0 (3)
C24—C15—C16—C21	−0.1 (5)	C14—C13—N1—C12	−42.4 (4)
C14—C15—C16—C21	−176.1 (3)	C3—C13—N1—C12	−163.8 (3)
C15—C16—C17—C18	−176.3 (3)	C11—C12—N1—C8	57.3 (4)
C21—C16—C17—C18	0.8 (5)	C11—C12—N1—C13	−175.7 (3)
C15—C16—C17—C13	−1.0 (4)	C5—C6—N2—C2	−57.1 (3)
C21—C16—C17—C13	176.2 (3)	C5—C6—N2—C1	179.1 (3)
N1—C13—C17—C18	56.5 (4)	C3—C2—N2—C6	73.3 (3)
C14—C13—C17—C18	174.9 (3)	C3—C2—N2—C1	−163.6 (3)
C3—C13—C17—C18	−61.0 (5)	C75—C74—O3—C77	−179.0 (4)
N1—C13—C17—C16	−118.0 (3)	C73—C74—O3—C77	2.5 (6)
C14—C13—C17—C16	0.4 (3)	C54—C55—O4—C58	−3.9 (6)
C3—C13—C17—C16	124.5 (3)	C56—C55—O4—C58	177.6 (4)
C16—C17—C18—C19	−3.0 (5)		

### Hydrogen-bond geometry (Å, º)

Cg1 is the centroid of the benzene ring (C52–C57) in the phenylmethylidene group.

**Table:**

*D*—H···*A*	*D*—H	H···*A*	*D*···*A*	*D*—H···*A*
C77—H77*A*···O2^i^	0.96	2.54	3.418 (5)	152
C58—H58*C*···*Cg*1^ii^	0.96	2.93	3.822 (5)	156

Symmetry codes: (i) −*x*+1, −*y*, −*z*; (ii) −*x*+2, −*y*, −*z*+1.
